# Combined application of virtual surgery and 3D printing technology in postoperative reconstruction of head and neck cancers

**DOI:** 10.1186/s12893-019-0616-3

**Published:** 2019-11-28

**Authors:** Chao Li, Yongchong Cai, Wei Wang, Yan Sun, Guojun Li, Amy L. Dimachkieh, Weidong Tian, Ronghao Sun

**Affiliations:** 1 Department of Head and Neck Surgery, Sichuan Cancer Hospital & Institute, Sichuan Cancer Center, School of Medicine, University of Electronic Science and Technology of China, No.55, 4th Section of Southern Renmin Road, Chengdu, 610041 Sichuan China; 2 Department of Otorhinolaryngology and Head and Neck Surgery, Yuhuangding Hospital of Qingdao University, Yantai, China; 3 Department of Head and Neck Surgery, The University of Texas MD Anderson Cancer Center, Houston, TX USA; 4 Department of Epidemiology, The University of Texas MD Anderson Cancer Center, Houston, TX USA; 5 Department of Pediatric Otolaryngology, Texas Children’s Hospital, Houston, TX USA; 6 Department of Otolaryngology – Head and Neck Surgery, Baylor College of Medicine, 6701 Fannin Street, Suite 540, Houston, TX 77030 USA; 7 Department of Oral and Maxillofacial Surgery, West China College of Stomatology, Sichuan University, Chengdu, China

**Keywords:** Head and neck cancer, Reconstruction, Digital surgery, Virtual reality, 3 dimensional printing, Computer aided design, Computer aided manufacturing

## Abstract

**Background:**

The complex anatomy of the head and neck creates a formidable challenge for surgical reconstruction. However, good functional reconstruction plays a vital role in the quality of life of patients undergoing head and neck surgery. Precision medical treatment in the field of head and neck surgery can greatly improve the prognosis of patients with head and neck tumors. In order to achieve better shape and function, a variety of modern techniques have been introduced to improve the restoration and reconstruction of head and neck surgical defects. Digital surgical technology has great potential applications in the clinical treatment of head and neck cancer because of its advantages of personalization and accuracy.

**Case presentation:**

Our department has identified the value of modern digital surgical techniques in the field of head and neck surgery and has explored its utility, including CAD/CAM technology and VR technology. We have achieved good results in the reconstruction of head and neck surgical resection defects.

**Conclusion:**

In this article, we share five typical cases from the department of head and neck surgery where the reconstruction was performed with the assistance of digital surgical technology.

## Background

The anatomy of head and neck is exceptionally complex. Numerous key organs, known vessels and named nerves converge here, leading to differences in tumors types and growth patterns. Only a complete resection of the tumor can improve survival, quality of life, and create conditions for safe adjuvant treatment. Patients often need to undergo radical resection for aggressive malignant tumors to ensure sufficient resection margins. Lack of effective reconstruction techniques in the distant past left doctors and patients with poor results and significant impacts on quality of life. With the development of microsurgical techniques, free tissue transfer reconstruction is now standard of care. In order to achieve complete resection and efficient and effective functional reconstruction, it is particularly important to develop a reasonable preoperative surgical plan and execute that plan in the operating room.

At present, conventional imaging techniques such as ultrasound, CT and MRI can reflect the relationship between the tumor and adjacent important tissues. When the soft tissue or osseous structure of the head and neck needs to be reconstructed after cancer extirpation, two-dimensional imaging can limit a comprehensive assessment and analysis of the disease. This makes preoperative planning difficult as it can be difficult to appreciate the surgical defect in three dimensions.

With the development of digital technology the possibility of better preoperative three dimensional planning became a reality. Virtual surgery (virtual reality and augmented reality), CAD, CAM (3D printing), RP, 3D navigation and robotics have been rapidly applied and combined to be used in a personalized approach making precision medicine possible in head and neck surgery. Among these, CAD, CAM and VR technology are the most commonly used [1, 2].

CAD technology uses CT or MRI imaging data to analyze 3D defects of anticipated extirpative surgery. This makes consideration of using RP by 3D printer to better model the resulting defect. This has led to a more personalized approach taking into consideration each individual patients unique requirements.

VR is a simulation of human-machine interface technology to generate a 3D virtual world. Using VR, a computer can create a realistic visual, auditory, and tactile sensory experience. VR technology has been widely used in military, industry, education and other fields. Arriving late in the field of medicine, it has emerged as a capable surgical assistant and opened a new model of preoperative assessment and surgical guidance. Applying VR technology to the head and neck can make complex structural relationships vivid and stereoscopic, making an abstract concept intuitive and clear, and bring great flexibility to the operative field. We recognize the importance of the application of CAD / CAM and VR technology in head and neck surgery. In order to improve outcomes of head and neck cancer patients with surgical treatment, we have applied these advances in our surgical practice. We present here our experience in a limited number of patients. The general clinical characteristics of all patients in this study is shown in Table 1.

**Table 1:** Characteristics of five patients in this study

Cases	1	2	3	4	5
Age (years)/sex	53/F	35/F	50/F	51/M	34/F
Pathology	Sarcoma	CA	ACC	SCC	ACC
Lesions	Neck	Neck	maxilla	mandible	mandible
Previous treatment	ERM + CT	No	RR + RT	No	No
Operative methods	ERPL+ PMMF	RR	ERPL +FND + FF	ERPL +RND + FF + AF	SM + FF
Main Digital Technology	VR	VR	CAD, CAM, VR, RP	3D, CAD, CAM, RP	CAD/CAM
Complications	No	Horner’s syndrome;	postoperative infection	No	No
Appearance	acceptable	acceptable	satisfactory	acceptable	satisfactory
Functional outcomes					
Diet	soft	solid	solid	soft	soft /liquid
Speech	normal	normal	intelligible	intelligible	intelligible
Motion of upper limb	Mild limitation	no limitation	no limitation	no limitation	no limitation
Follow-up (months)	19	17	69	51	24
Status	AWD	AND	AND	AND	AND

*F* female, *M* male, *ACC* adenoid cystic carcinoma, *SCC* squamous cell carcinoma, *CA* carotid aneurysm, *CT* chemotherapy, *RT* radiotherapy, *FND* functional neck dissection, *RND* radical neck dissection, *RR* radical resection, *ERPL* enlarged resection of primary lesions, *MFF* myocutaneous free flaps, *FF* fibula flap, *LF* Iliac bone flap, *PMF* pectoralis major flap, *AF* adjacent flaps, *PMMF* Pectoralis major muscle flap, *ERM* extensive radical mastectomy, *SM* segmental mandibulectomy, *VR* virtual reality, *3D* three dimensional, *CAD* computer aided design, *CAM* computer aided manufacturing, *RP* rapid prototyping, *AR* augmented reality; Functional outcomes [diet (solid, soft, liquid, or nasogastric tube feeding), speech (normal, intelligible, slurred, or requirement for a tracheostomy), and range of motion of the upper limb (severe limitation, moderate limitation, mild limitation, no limitation)]; *AWD* alive with disease, *AND* alive with no disease

## Case presentation

### Case 1

**Fig. 1:**
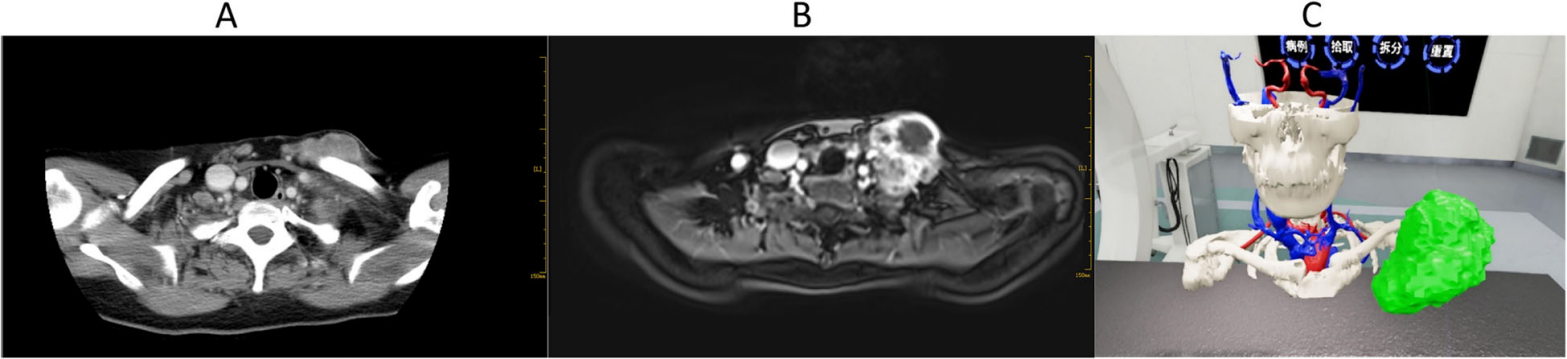
**a** CT shows the range of tumor invasion (cross section); **b** MRI shows the range of tumor invasion (cross section); **c** Surgical simulation which using gestures to pick up, rotate, zoom, model resolution, profile and other operations by VR technology

A 53 year-old female presented with a left neck mass. Her past medical history is significant for recurrent breast cancer that required multiple surgeries including radical mastectomy and two courses of chemoradiotherapy. Three years prior to presentation, a mass was found in her left neck. A needle biopsy demonstrated malignant fibrous histiocytoma. Imaging with CT and MRI were obtained and shown in Fig. 1a, b. Following preoperative physical examination and imaging, VR was used to simulate the operation. The CT and MRI data were collected and converted into DICOM format. The DICOM image was tested to ensure that the image meets quality standards. The DICOM images were used to perform a three dimensional reconstruction of the area of anticipated resection. Multimodal image fusion of CT and MRI was performed using precision detection by medical imaging experts. The multimodal 3D reconstruction model was transformed into VR model, with color coding of important structures, blood vessels, nerves, bone and tumor for convenient recognition. This model is imported into the UE4 engine to take advantage of function customization. After settling cluster rendering equipment and 3D scanner, surgeons wear head mounted display and data gloves for surgical simulation to pick up and rotate the model, or to change functions such as zoom, model resolution, and profile in the virtual operation room (Fig. 1c). Following use of the VR with multiple sessions, the patient underwent extended excision of left cervical and thoracic junction tumor with partial left clavicle resection, left subclavian vein repair, and pectoralis major myocutaneous flap repair under general anesthesia.

### Case 2

**Fig. 2:**
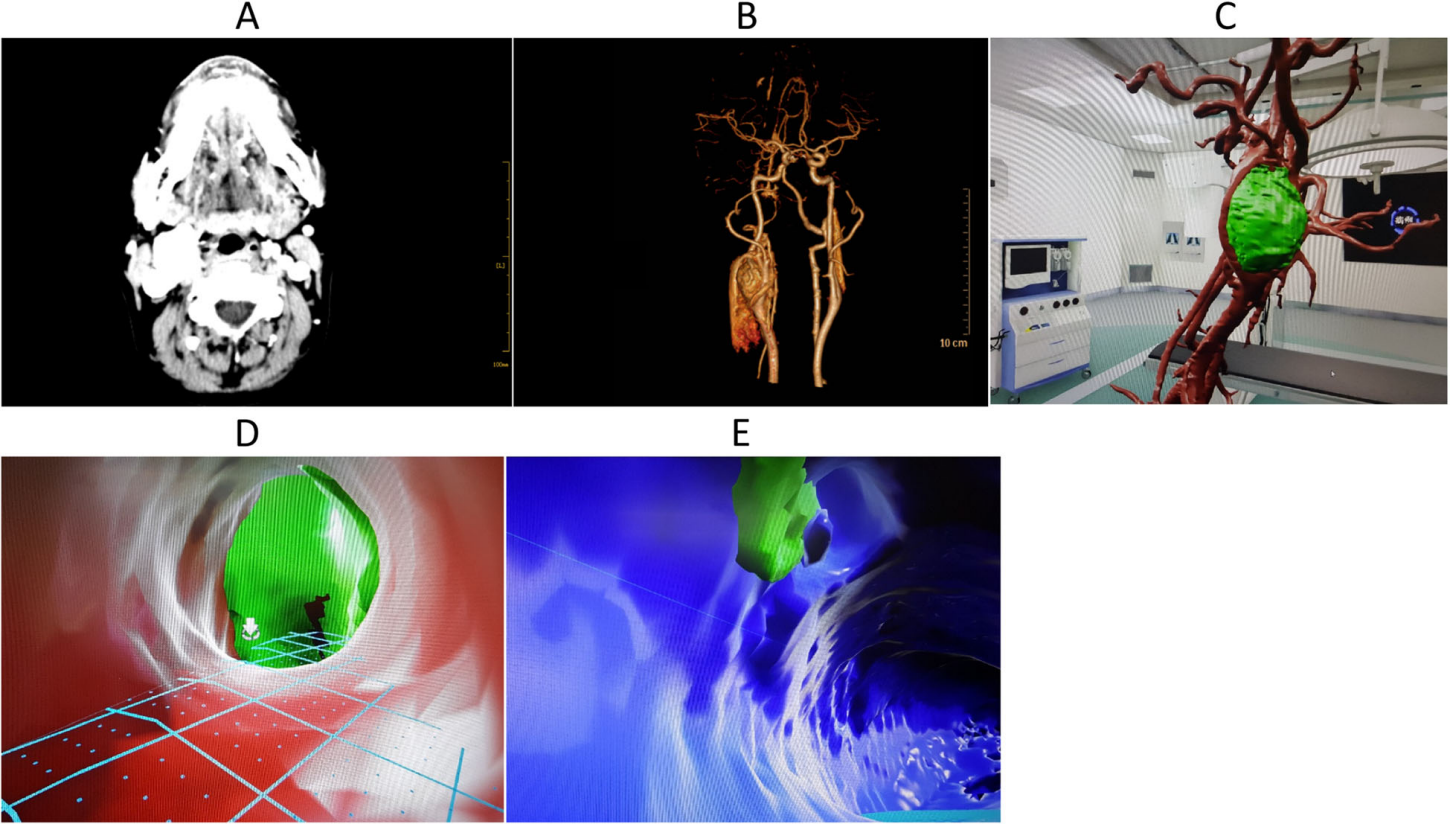
**a** CT shows the relationship between tumor and adjacent tissue; **b** CTA shows the relationship between tumor and blood vessel; **c** VR model after removal of the venous system; **d** Intravascular peep from the internal arteries of the carotid body tumor; **e** Intravascular peep from the vein of the carotid body tumor

A 35 year-old female presented with a right neck mass that she noticed 5 months previously. The tumor was approximately 3 × 3 cm and was initially discovered due to a work-up for a stroke. A contrast-enhanced neck CT showed a 2.5 × 2.5 cm tumor at the right carotid artery bifurcation (Fig. 2a). The tumor surrounded the initial segment of internal and external carotid artery, with circumferential collateral vessels consistent with a carotid body tumor. CTA confirmed right common, internal and external carotids supplying the tumor (Fig. 2b). Preoperative VR technique was used to make individual models and to perform preoperative simulation of the anticipated resection (Fig. 2c). The unique perspective of using VR technology to view the blood vessels, called “intravascular peep”, clearly demonstrates vascular compression or vascular invasion (Fig. 2d, e). After optimizing the preoperative planning, the patient underwent right carotid body tumor resection. VR allowed the surgeon to practice the gradual separation of the tumor at the carotid bifurcation.

### Case 3

**Fig. 3:**
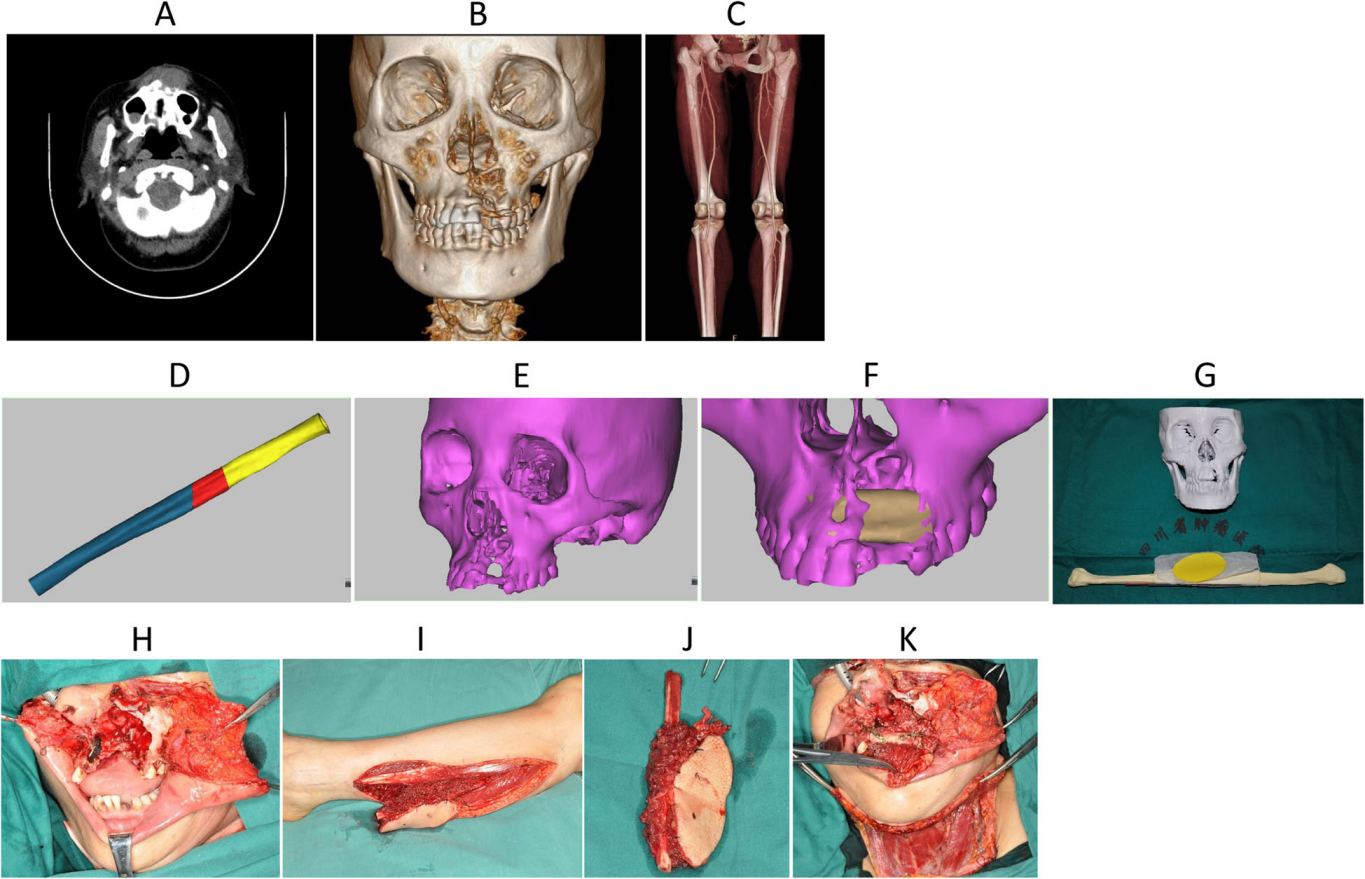
**a** CT transect showed that the lesion infiltrated into the left vestibule area, involving the nasal septum and the nasal floor; **b** Three-dimensional reconstruction of maxillofacial region, bone defect; **c** and lower extremity vessels by CAD technique after CT angiography; **d** Computer simulation for repair of maxillofacial region; **e** The position, length, arc of the fibula and the angle of the osteotomy of the fibula used by computer simulation and repair; **f** The effect of computer simulation after repair; **g** Three-dimensional printers’ rapid prototyping model; **h** The left maxillary tumor resection (resection including the left maxillary sinus wall, inferior wall, anterior wall, the section on the right side of the maxillary sinus and inferior wall, by simultaneous resection of nasal septum and nasal tumor infiltrating the bottom); **i** The skin flap was designed as the center of the skin before operation, and the skin of the left calf was cut into the perforator to dissect the perforating branch of the peroneal artery; **j** Vascularized free fibula myocutaneous flap was made by truncated fibula; **k** Repair effect of vascularized free fibula myocutaneous flap during operation

A 50 year-old female with history of adenoid cystic carcinoma status post partial maxillectomy 3 years ago, presented with recurrence in the left maxilla. A 5.0 × 4.5 cm mass was palpable in left cheek and she underwent radiation therapy with minimal to no response. When the patient presented our service, she underwent preoperative examination and imaging. Maxillofacial contrast-enhanced CT (Fig. 3a) and CT three-dimensional reconstruction of the lesion and of the lower extremity vessels was performed by CAD technique (the 3-dimensional reconstruction technology and virtual surgery) after CT angiography (Fig. 3b, c). Before surgery, a rapid prototyped template was manufactured to help determine the planned lengths and angles of the fibula osteotomies and to guide the insertion of dental implants, which were placed in the free fibula graft prior to resection. In addition, a premanufactured infraorbital implant was planned for insertion to compensate for the patients infraorbital and zygomatic bony defect. The simulated reconstruction was modeled on the computer (Fig. 3d-f) with rapid prototyping by 3D printer (Fig. 3g). The patient then underwent excision of the maxillary tumor, nasal septal resection, bilateral neck dissection, fibula myocutaneous flap repair, and abdominal free skin grafting. We use a 3D printed osteotomy plate after CAD design to precisely perform the appropriate osteotomies for resection of the tumor. A pre-bent titanium plate and screw were used to fix the jaw. The precise nature of respectable osteotomy and preplanned pre-bent plate allowed for a more personalized reconstruction.

### Case 4

**Fig. 4:**
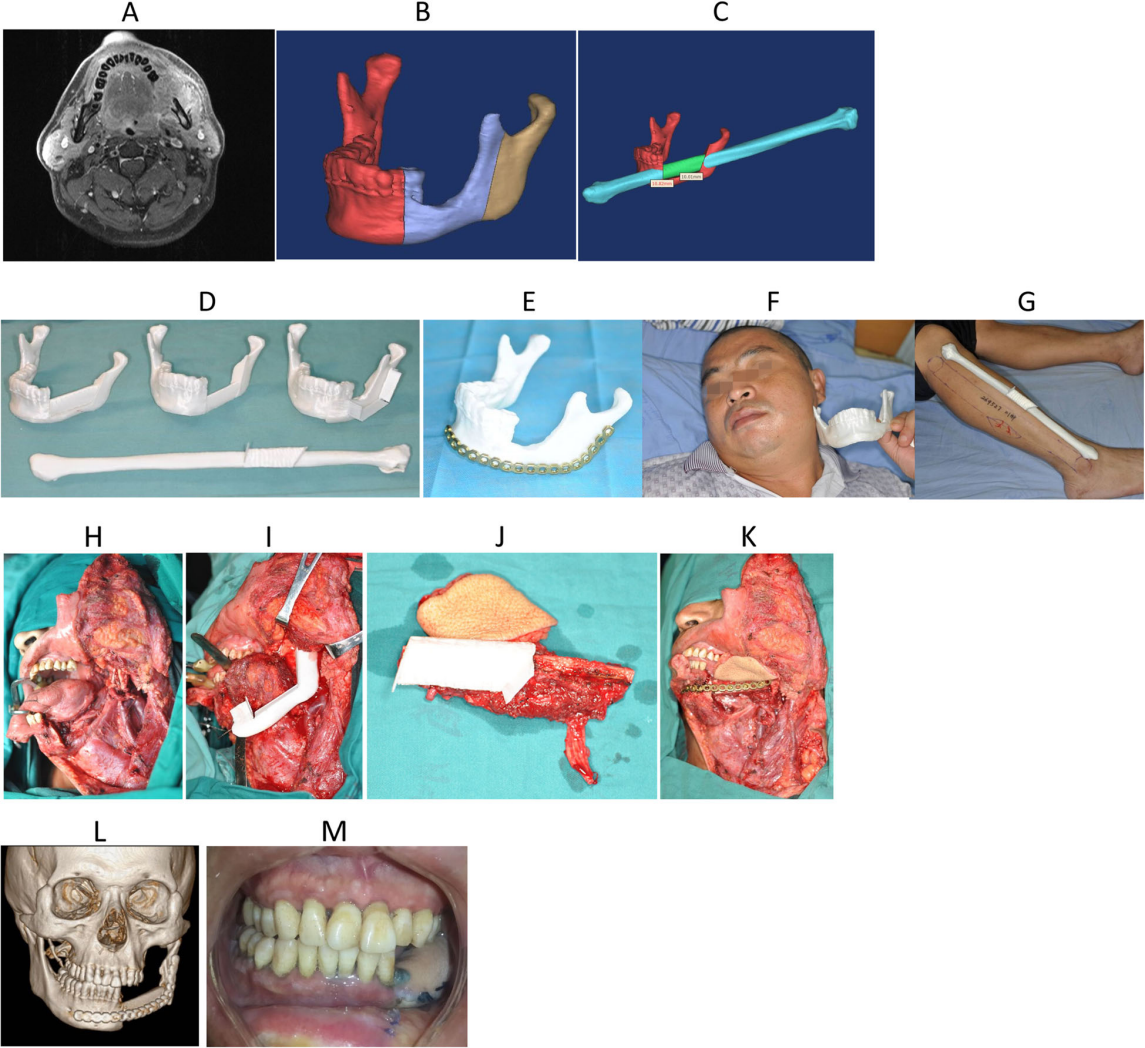
Preoperative performance and computer simulation of patients, model of rapid prototyping by 3D printer, one-stage repair of mandibular defect by CAD/CAM technique, and the follow-up of CAD/CAM assisted individualized repair of complex segmental defects in mandible. **a** The scope of invasion (transverse section); **b** The range of simulated surgical excision; **c** Osteotomy range and repair of simulated fibula flap; **d** Customize the osteotomy plate according to the model after the rapid prototyping, determine the interception range and location of the fibula; **e** Pre-bending of titanium plate according to the model after rapid prototyping; **f** The condition of mandible defect in patients with equal proportions; **g** The right fibula and osteotomy area of the equal proportions of the patients. **h** Expanded resection of tumor shows the area to be repaired; **i** The range of segmental resection of the mandible in the process of enlarged tumor resection is consistent with the preoperative simulation; **j** Preparation of free fibula flap according to the osteotomy plate model; **k** Repair of the defect area with free fibula flap and fixed with preformed titanium plate; **l** Three dimensional reconstruction of CT scan in patients with postoperative lesions and repair and reconstruction; **m** The degree of occlusion was good and the function of the temporomandibular joint was normal

A 51 year-old male presented with a 2 month history of a 2.5 × 3.5 cm left gingival squamous cell carcinoma. On physical exam, the left mandible was involved as were multiple ipsilateral lymph nodes. The largest of which was 3 × 3 cm. The contrast-enhanced MRI scan in the maxillofacial region is shown in Fig. 4a. We used CAD/CAM technology to assist with surgical planning (Fig. 4b, c), then we used plastic and a resin composite material for rapid prototyping through the 3D printer (Fig. 4d-g). We developed an equiratio 3D anatomical model and osteotomy plate. The patient underwent segmental mandibulectomy with free fibula reconstruction (Fig. 4h). The fibula osteotomies on the pedicle vascularized free fibular flap were cut according to the preoperative simulation (Fig. 4g). A custom pre-bent plate was designed according to the computer simulation and the 3D model. We fixed the custom titanium plate at a predetermined position according to the simulation data and 3D model, and the oral defect was simultaneously repaired by the soft tissue component of the flap (Fig. 4i, k).

Post-operatively the reconstruction did well. The patient was followed up with good facial morphology and postoperative CT imaging showed good contour of the fibula. The fixation position of titanium plate screws were accurate (Fig. 4l), and the occlusion was normal (Fig. 4m).

### Case 5

**Fig. 5:**
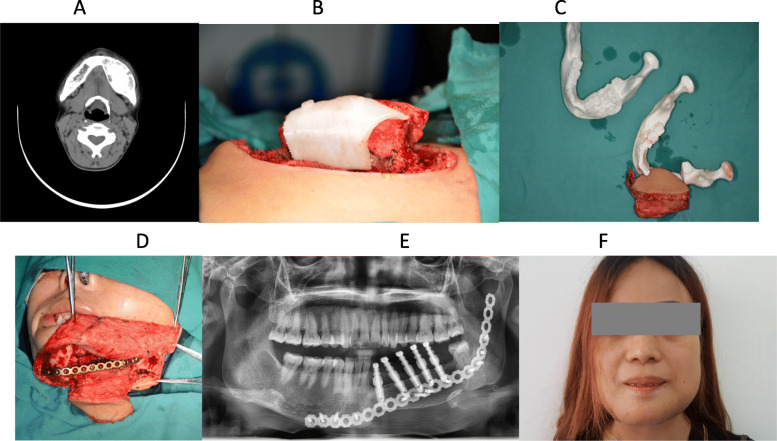
**a** The CT in the maxillofacial region shown that the bone enlargement, destruction, and irregular mass of the mandible with a larger scope of approximately 5.5*3.1 cm; **b** Preparation of vascularized free iliac musculocutaneous flap; **c** Comparison of the prepared iliac bone flap with the 3D model; **d** Use of prefabricated titanium plates for fixing the disconnected mandible and the intercepted iliac bone; and **e** The second stage of dental implants; **f** The facial appearance and occlusion function after follow-up

A 34 year-old woman presented with a left mandibular mass that had been present for approximately 5 months. Physical examination demonstrated a 5 cm left submucosal oral cavity lesion with clinically evident mandibular expansion and erosion. CT imaging demonstrated a 5.5 × 3.1 cm mandibular lesion. Several small lymph nodes were also shown on the both sides of the neck, the larger diameter of which was approximately 0.7 cm (Fig. 5a). A biopsy of the left mandibular mass showed fibrous lesions of bone which inclined to cementite fibroma.

We used CAD/CAM technology to develop a surgical treatment plan. Based on the tumor size and areas of invasive disease, segmental mandibulectomy was simulated. We modeled the simulated cut with the iliac bone in the defect area in the width, thickness, and angle. The place for osteotomy of the iliac bone was determined, and the virtual reconstruction was performed. A three dimensional solid model, osteotomy template of the lesion, and the iliac bone were developed through a 3D printer. Pre-bent titanium plates were prepared by the reconstructive model of rapid prototyping.

Following the simulated surgery, the left mandibular segmental resection with free iliac myocutaneous flap reconstruction with titanium plate fixation were performed under the general anesthesia. (Fig. 5b, c). Prefabricated titanium plates were used to fix the iliac bone to the mandibular resection defect (Fig. 5d). The defect in the oral cavity was repaired with the muscle flap. During the follow-up, the second stage of dental implants were placed 1.5 years after the first stage of operation (Fig. 5e). Currently the patient had a nice facial appearance with an excellent occlusion, and a functional oral cavity (Fig. 5f).

## Discussion and conclusions

With the rapid development of medical imaging digitalization, computer assisted surgical simulation based on the 3D reconstruction have developed rapidly. 3D reconstruction of complex head and neck cancers can elucidate important information such as tumor location, scope of invasion, blood supply, and also provide the potential for preoperative simulation for complex defect repair. In the past, “three dimensional” imaging still depended on a “one-dimensional” modalities. The advent of high-precision 3D printers and VR technologies have significantly changed the potential of virtual visualization. After a simple post-processing and format conversion, the reconstructed image can directly produce a vivid and precise physical model.

In the early 1990s, scholars began to apply CAD and 3D printing techniques to the diagnosis and treatment of complex head and neck and maxillofacial diseases. These two techniques improved diagnostic accuracy by 29.60%, procedural precision by 36.23%, and shortened operative time by 17.63% [3]. In the twenty-first century, CAD and 3D printing techniques have been widely used for reconstruction of complex defects in head and neck surgery [4, 5]. These techniques provide an accurate preoperative simulation and intraoperative surgical ablative plan. Additionally, enhanced imaging provides a reference for the length and shape of fixed titanium plates and screw positions for osseous reconstruction. In recent years, some scholars have pioneered the comprehensive application of CAD combined with 3D printing technology to guide free vascularized fibula free flap reconstruction of maxillary defects [6, 7]. Some scholars have also taken the lead in introducing VR technology in the development of operative planning and pre-operative simulation [8].

Although CAD/CAM and VR technology has a wide range of potential applications in ablative surgical planning and complex reconstruction of head and neck cancer, it is still in the exploratory stage. In China, the above technologies are available in many units or companies with required hardware and software. Our department has accumulated some experience in this field. We concede that the application of these technologies increases the cost of treatment and extended surgeon time in preparation. We believe, however, that the cost and time can be reduced in a center that has the expertise and can use the technology in batches. At present, the annual application of these digital technologies in our center is about 30–50 cases per year. According to our application experience, preoperative preparation time needs about 2–4 days, and we feel it is an acceptable delay for both the patient and the surgeon. In recent years, some studies have suggested that use of digital technology does not significantly increase the cost of treatment, and has obvious benefits for multi-segment osteotomy cases [9]. We have identified several advantages using this technology: 1) the surgical margin of the tumor can be determined according to the invasion of solid tumor and the anatomical characteristics of the patient maximizing preservation of maxillofacial bone tissue with oncological possibility; 2) the personalized 3D-printed bone model and osteotomy plates are convenient for surgeons to visualize tumors preoperatively to confirm the location and extent of the tumor, location, and length, as well as the angle of the osteotomies, which can improve three-dimensional repeatability [10]; 3) in the case of severe osseous deformity caused by tumor invasion, the data from the contralateral normal mandible can be collected and inverted by mirror image technology. After setting up the mirror model, the titanium plate is pre-bent with preset plate and screw positioning for the best fixation. During the operation, to maximize oral cavity function and cosmetic results, a plastic drill guide can be used as a template for harvesting an exact fibula construct with precise osteotomies for detailed reconstruction [6]. CAD/CAM group has advantages in function and aesthetic achievement [11]; 4) these 3D-models can be used preoperatively for modeling titanium mesh to repair large mandibular defects, or even to directly manufacture personalized titanium mandibular prostheses for immediate reconstruction [12].; and 5) patient-specific models can improve doctor-patient communication. These modes are convenient for patients to understand the details of the operation, predict the cosmetic effects of surgery, and understand the possible risks during perioperative planning, and may help improve patient compliance.

Our department has made full use of the various applications of CAD/CAM techniques to assist in the reconstruction of the segmental defect of the mandible and maxilla (for example case 4). A complete set of digital techniques was used to simulate the operation to determine the scope of the procedure and to design the shape of the osseous graft. We used virtual practice to predict the possible difficulties in the operation and fine tune the surgical plan (Fig. 4b, c). We strive to determine the best surgical procedure and optimize cosmetic and oncologic outcomes with enhanced surgeon-patient communication prior to the operation. Personalized modeling of the bone, the planned ablative defect and the reconstruction with titanium plate (Fig. 4d-g) makes surgical planning more accurate with less guess work during the operation. It also reduces operative time and postoperative complications. Additionally, it optimizes postoperative reconstruction cosmetics by contouring the titanium plate and positioning screws in best fixed position by digital technology (Fig. 4i, k, m).

After success with multiple applications of CAD/CAM technology in the reconstruction of head and neck defects, our department is expanding applications of VR technology to visually explore human anatomy. Previous studies have shown that VR technology is not only a good way of simulating surgery for surgeons [13], but also enables surgeons to better understand the relationship between surgical approaches and adjacent structures to improve surgeon proficiency and reduce unanticipated complications during surgery [14, 15].

In our experience, when compared with previous methods of two-dimensional preoperative assessment, surgeries using VR technology require more complex and time-intensive preoperative assessment. However, we feel there are more advantages to this approach for the reconstruction of complex head and neck defects. VR technology can provide morphological and functional information by simultaneously combining the advantages of CT in bone invasion with MRI in soft tissue involvement by tumor [16]. By building a virtual stereoscopic medical image, a realistic 720 degree 3D image model provides the surgeon with the most complete preoperative assessment. The headgear and data gloves can be used to adjust the size and direction of the field of vision for an immersive operative experience. When a surgeon performed the simulated operation, multiple assistants can interact by wearing a device that allows the entire medical team to become familiar with the patient’s condition and improve operative coordination. Patients can participate in the surgical process, experience the simulation, understand the disease, and optimize patient-physician communication. Moreover, the use of VR technology does not require material input or capital investment.

In unique cases, such as the carotid body tumor above, when both imaging and virtual images suggest that the tumor is closely linked to the blood vessels, VR technology can allow the physician visualize the intraluminal dimension. Based on a CT or MRI angiogram the involvement of the vessel can be anticipated (Fig. 2d, e). This is particularly useful in cases of post-radiation imaging when tissues are significantly fibrotic and exposing vascular anatomy can be challenging. Preoperative knowledge of vascular invasion can help surgeons anticipate possible need for increased operative time or vascular surgeon consultation.

To improve oral cavity function digital surgery can more precisely anticipate the resection with wide surgical margins and customize fibular free flap reconstruction in oral cavity tumors with mandibular invasion.

We conclude that computer-assisted surgery for personalized reconstruction of complex defects of the head and neck has role in clarifying tumor anatomy relationships, reconstructing complex osseous and soft tissue defects and defining vascular lesions such as aneurysms and vascular tumors. This information leads to precise surgical treatment of head and neck cancer patients. However, its application in head and neck surgery is still limited. More systematic clinical results are needed to confirm the overall and reliable clinical value. Nonetheless, we believe that using computer-aided digital surgical technology to evaluate, simulate, formulate, and implement operative plans is an important trend in the future of head and neck surgery.

## Data Availability

Other data is available upon request from the corresponding authors as long as it does not cause the identification of the patient or violate any institutional law regarding data confidentiality.

## Abbreviations

3D: Three dimensional
AR: Augmented reality
CAD: Computer aided design
CAM: Computer aided manufacturing
RP: Rapid prototyping
VR: Virtual reality
